# Molecular Relapse After a Second Treatment‐Free Remission Attempt Following Asciminib in Chronic Myeloid Leukemia: A Case Report

**DOI:** 10.1155/crh/1621284

**Published:** 2026-06-27

**Authors:** Nobue Sato, Ryo Yoshimaru, Yong-Mei Guo, Hirotaka Nakamura, Kensuke Matsuda, Yosuke Minami, Junichiro Yuda

**Affiliations:** ^1^ Department of Pharmacy, National Cancer Center Hospital East, Kashiwa, Japan, ncc.go.jp; ^2^ Department of Hematology and Oncology, National Cancer Center Hospital East, Kashiwa, Japan, ncc.go.jp

**Keywords:** asciminib, case report, chronic myeloid leukemia, molecular relapse, treatment-free remission, tyrosine kinase inhibitors

## Abstract

Treatment‐free remission (TFR) has become an important therapeutic goal in chronic myeloid leukemia (CML). Although the outcomes of TFR following adenosine triphosphate (ATP)–competitive tyrosine kinase inhibitors (TKIs) have been established, data on TFR after asciminib discontinuation remain limited. In this study, we report the case of a man in his 60s with chronic‐phase CML who experienced molecular relapse 8 months after a second TFR attempt following asciminib treatment. The patient was diagnosed with CML approximately 15 years earlier and achieved deep molecular response 4.5 (MR4.5), representing a 4.5‐log reduction in *BCR::ABL1* transcripts, with first‐line imatinib. After the first TFR attempt, major molecular response (MMR) was lost, and ponatinib was initiated but discontinued because of cerebral infarction. Asciminib was subsequently introduced as third‐line therapy at a dose of 40 mg twice daily, and *BCR::ABL1* transcripts remained undetectable for more than 3 years. Based on the sustained deep molecular response and patient preference, asciminib was discontinued. Eight months after discontinuation, however, loss of MR4.0 (BCR::ABL1 > 0.01% IS) was detected. This threshold was used to guide treatment reinitiation, rather than waiting for loss of MMR as recommended by the ELN guidelines, in the view of the clinical context of a second TFR attempt following prior failure. Imatinib was reintroduced, leading to the reachievement of a deep molecular response. This case underscores the importance of careful patient selection and long‐term molecular monitoring following TFR attempts, including those involving novel TKIs such as asciminib.

## 1. Introduction

Treatment‐free remission (TFR) has emerged as an important therapeutic goal aiming to reduce long‐term treatment‐related toxicity and healthcare costs in chronic myeloid leukemia (CML). Current European LeukemiaNET guidelines recommend that selected patients with chronic‐phase CML who exhibit stable deep molecular response (DMR) can be considered for tyrosine kinase inhibitor (TKI) discontinuation under close molecular monitoring [1]. Using established eligibility criteria, approximately 50% of the patients who discontinue TKI therapy can sustain TFR, whereas the remaining patients experience molecular relapse, most commonly within the first 6 months after treatment cessation [2].

The evidence supporting TFR strategies was largely derived from studies involving adenosine triphosphate (ATP)–competitive TKIs, including imatinib, dasatinib, nilotinib, and bosutinib. By contrast, data on TFR following asciminib discontinuation remain limited. Asciminib is a first‐in‐class agent that targets the myristoyl pocket of the BCR::ABL1 oncoprotein rather than the ATP‐binding site, distinguishing it mechanistically from conventional TKIs [3]. This unique mechanism has been associated with favorable molecular response and improved tolerability, particularly in patients with prior TKI intolerance or resistance [4].

To date, few studies describing TFR after asciminib discontinuation have been reported. Yoshimaru and Minami [5] described four patients with CML who exhibited increased *BCR-ABL1* transcript levels after asciminib cessation, with three patients regaining DMR after treatment reinitiation. Ernst et al. [6] reported a patient carrying the T315I mutation who maintained TFR for 18 months following asciminib discontinuation. More recently, Yousefi et al. [7] described two patients who sustained TFR for at least 8 months after treatment discontinuation without molecular relapse. In this context, we report a case of molecular relapse occurring after a second attempt at TFR following asciminib therapy, highlighting the challenges of durable TFR with novel TKIs and the need for sustained molecular surveillance even in patients with prolonged DMR.

## 2. Case Presentation

A man in his 60s with essential hypertension and Type 2 diabetes mellitus was diagnosed with chronic‐phase CML approximately 15 years prior to presentation. The Sokal score at diagnosis indicated low‐risk disease, and treatment was initiated with imatinib 400 mg once daily. The patient achieved and maintained DMR 4.5 (MR4.5), denoting a 4.5‐log reduction in *BCR::ABL1* transcripts, for approximately 10 years.

As both the duration of DMR and total TKI treatment met the established criteria for TFR, imatinib was discontinued following discussion with the patient. Approximately 3 months after discontinuation, *BCR::ABL1* transcript levels increased to 0.2251% on the International Scale (IS), indicating loss of major molecular response (MMR). Ponatinib was initiated at 15 mg once daily, resulting in a decrease in *BCR::ABL1* transcripts to 0.0081% IS (MR4.0 or deeper). The dose was subsequently increased to 30 mg once daily; however, the patient developed acute cerebral infarction, necessitating immediate discontinuation of ponatinib.

After recovery, molecular relapse was observed with loss of MMR (0.1635% IS). Asciminib was then initiated at a dose of 40 mg twice daily. From 6 months onward, *BCR::ABL1* transcripts became undetectable (< 0.0007% IS), and DMR (MR4.5 or deeper) was maintained for approximately 3 years.

At this stage, the total duration of TKI therapy was approximately 14 years, and the duration of DMR under asciminib was approximately 3 years. The patient strongly desired TKI discontinuation to reduce both his economic and physical treatment burden. After thorough discussion of the risks of TFR failure and obtaining informed consent, asciminib was discontinued.

**FIGURE 1:**
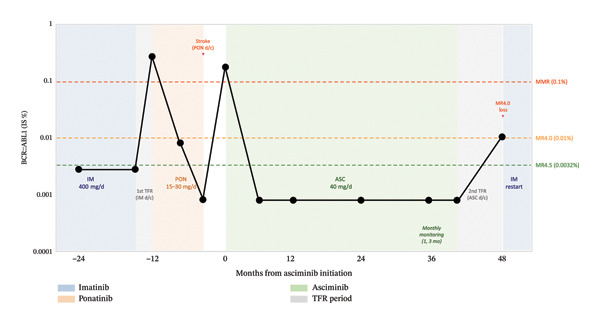
Treatment course and longitudinal changes in *BCR::ABL1* transcript levels (IS %). The *x*‐axis presents time relative to asciminib initiation (Month 0). Colored backgrounds represent treatment periods: blue, imatinib; orange, ponatinib; green, asciminib; gray, treatment‐free remission periods. Dashed horizontal lines denote molecular response thresholds: MMR (0.1%), MR4.0 (0.01%), and MR4.5 (0.0032%). Abbreviations: CML, chronic myeloid leukemia; DMR, deep molecular response; IS, international scale; MMR, major molecular response; MR4.0, deep molecular response 4.0; MR4.5, deep molecular response 4.5; TFR, treatment‐free remission; TKI, tyrosine kinase inhibitor.

Molecular monitoring was performed monthly. Eight months after asciminib discontinuation, loss of MR4.0 was detected, with *BCR::ABL1* transcripts exceeding 0.01% IS. Imatinib 400 mg once daily was reinitiated, resulting in prompt reachievement of MR4.5 or deeper, which has been maintained to date (Figure 1). No TKI withdrawal syndrome was observed following asciminib discontinuation.

## 3. Discussion

This case describes molecular relapse occurring 8 months after a second attempt at TFR following asciminib therapy. To our knowledge, reports describing TFR outcomes specifically after a second TFR attempt following asciminib therapy are scarce, making this case a clinically relevant addition to the existing literature. Prior reports by Yoshimaru and Minami [5], Ernst et al. [6], and Yousefi et al. [7] have described TFR outcomes after asciminib discontinuation; however, none of these cases involved a second TFR attempt, underscoring the novelty of the present report. Established factors associated with successful TFR include the total TKI treatment duration, DMR maintenance duration, type of TKI used, and baseline Sokal risk score [2, 8, 9]. In the present case, the patient had a low Sokal score, a prolonged total TKI treatment duration of approximately 14 years, and sustained DMR for approximately 3 years prior to asciminib discontinuation, all of which exceeded commonly recommended thresholds for TFR eligibility. However, the first TFR attempt was unsuccessful. The success rate of second TFR attempts remains controversial. Legros et al. [10] reported TFR rates of 48%, 42%, and 35% at 12, 24, and 36 months, respectively, suggesting lower success rates compared with that of the first attempt. By contrast, the prospective DAstop2 study identified TFR rates of 61%, 56%, and 46% at 6, 12, and 24 months, respectively, indicating comparable outcomes as the first attempt [11]. These findings highlight the ongoing uncertainty surrounding repeat TFR strategies.

Evidence regarding TFR outcomes following asciminib discontinuation remains limited. Ernst et al. [6] reported sustained TFR for 18 months after 7.3 years of asciminib therapy in a patient carrying the T315I mutation. Yousefi et al. [7] described two patients who maintained TFR for at least 8 months after asciminib discontinuation. Both patients were intolerant, but not resistant, to ATP‐competitive TKIs, which might have contributed to favorable outcomes. By contrast, Yoshimaru and Minami [5] reported increased *BCR::ABL1* transcript levels in four patients after asciminib discontinuation although three patients reachieved DMR upon treatment reinitiation; notably, all patients in that series had a history of TKI resistance. Unlike conventional ATP‐competitive TKIs, asciminib exerts its inhibitory effect by binding to the myristoyl pocket of *BCR-ABL1*, a mechanistically distinct binding site [3]. Whether this unique mode of action influences TFR outcomes—for example, through differential effects on leukemic stem cell quiescence or immune surveillance—remains to be clarified. Prospective studies specifically designed to evaluate TFR following asciminib therapy are warranted to address this question.

In the present case, the patient had neither a T315I mutation nor documented TKI resistance, yet molecular relapse occurred 8 months after asciminib discontinuation, beyond the period when most relapses are typically observed. Notably, during the patient’s first TFR attempt, molecular relapse occurred as early as 3 months after imatinib discontinuation, whereas relapse was not detected until 8 months after the second TFR attempt following asciminib therapy. Whether this difference reflects the distinct pharmacological properties of asciminib, the longer duration of DMR prior to the second discontinuation, or other patient‐specific factors remains unclear. This finding underscores the importance of continued long‐term molecular monitoring, even in patients undergoing TFR attempts with asciminib and those with prolonged DMR.

It is also notably that treatment reinitiation in this case was prompted by loss of MR4.0 (BCR::ABL1 > 0.01% IS), a more conservative threshold than the loss of MMR (BCR::ABL1 > 0.1% IS) recommended by the ELN guidelines as the standard criterion for restarting therapy. This approach was chosen deliberately, given the clinical context of a second TFR attempt following an earlier failure. Because the first TFR attempt had been unsuccessful, a cautious strategy with earlier intervention was considered appropriate to prevent progression to deeper molecular relapse. This case underscores that the definition of molecular relapse prompting treatment reinitiation may need to be individualized, based on clinical circumstances, and that a conservative threshold can be justified in selected scenarios, particularly those undergoing a second TFR attempt.

This report was limited by its single‐patient nature, and its conclusions should, therefore, be interpreted with caution. In addition, this case involved a second TFR attempt following prior failure, which might not be directly comparable to the first attempt at TFR.

In conclusion, we described a case of molecular relapse occurring 8 months after a second TFR attempt following asciminib therapy. This case adds to the limited but expanding body of literature on TFR after asciminib therapy and, to our knowledge, is the first to document molecular relapse following a second TFR attempt in this context. These findings highlight the need for sustained molecular surveillance after asciminib discontinuation and support the need for prospective studies to better define eligibility criteria and monitoring strategies for TFR with asciminib.

## Author Contributions

Nobue Sato and Junichiro Yuda wrote the first draft of the manuscript. Ryo Yoshimaru, Yong‐Mei Guo, Hirotaka Nakamura, Kensuke Matsuda, and Yosuke Minami revised and approved the final version of the manuscript. Junichiro Yuda had full access to all study data and assumes full responsibility for the integrity of the data and the accuracy of the analysis.

## Funding

No funding was received for conducting this work.

## Disclosure

All authors have read and approved the final version of the manuscript.

## Ethics Statement

This study was conducted in accordance with the Declaration of Helsinki. Given the retrospective and observational nature of this single case report, formal ethics committee approval was not required according to institutional guidelines.

## Consent

Written informed consent was obtained from the patient included in this study.

The patient has consented to the submission of the case report to the journal.

## Conflicts of Interest

Nobue Sato, Ryo Yoshimaru, Yong‐Mei Guo, Hirotaka Nakamura, and Kensuke Matsuda declare no conflicts of interests. Yosuke Minami has received honoraria from Bristol‐Myers Squibb, Novartis, Pfizer, Daiichi Sankyo, and Takeda, and research funding from Ono Pharmaceutical, CMIC, Bristol‐Myers Squibb, Takeda, and Chugai Pharmaceutical. Junichiro Yuda has received research funding from AbbVie, Daiichi Sankyo, Chugai Pharmaceutical, Genmab, Novartis, Amgen, Takeda, Bristol‐Myers Squibb, Incyte, Janssen, Sumitomo Pharma, MSD, and Mitsubishi Tanabe Pharma.

## Supporting Information

Additional supporting information can be found online in the Supporting Information section.

## Supporting information


**Supporting Information** This case report has been prepared in accordance with the CARE guidelines. The CARE checklist is provided as supporting information.

## Data Availability

Data sharing is not applicable to this article as no datasets were generated or analyzed during the current study.
