# Supplementary material for: Molecular Relapse After a Second Treatment‐Free Remission Attempt Following Asciminib in Chronic Myeloid Leukemia: A Case Report
Source: Case Rep Hematol. 2026 Jun 27;2026:1621284. doi: 10.1155/crh/1621284 (PMC13309912; doi:10.1155/crh/1621284)
Supplement: Supplementary file 1 — Supporting Information This case report has been prepared in accordance with the CARE guidelines. The CARE checklist is provided as supporting information. [file CRH-2026-1621284-s001.docx]

**CARE Checklist of information to include when writing a case report**

**Manuscript:** Molecular Relapse After Second Treatment-Free Remission Attempt Following Asciminib in Chronic Myeloid Leukemia: A Case Report

| **Topic** | **Item** | **Checklist item description** | **Reported on Line/Page** |
| --- | --- | --- | --- |
| **Title** | 1 | The diagnosis or intervention of primary focus followed by the words "case report" | Page 1 |
| **Key Words** | 2 | 2 to 5 key words that identify diagnoses or interventions in this case report, including "case report" | Page 1 |
| **Abstract** | 3a | Introduction: What is unique about this case and what does it add to the scientific literature? | Page 1 (Abstract) |
|  | 3b | Main symptoms and/or important clinical findings | Page 1 (Abstract) |
|  | 3c | The main diagnoses, therapeutic interventions, and outcomes | Page 1 (Abstract) |
|  | 3d | Conclusion—What is the main "take-away" lesson(s) from this case? | Page 1 (Abstract) |
| **Introduction** | 4 | One or two paragraphs summarizing why this case is unique (may include references) | Pages 1-2 (Introduction) |
| **Patient Information** | 5a | De-identified patient specific information | Page 2 (Case Report, para 1) |
|  | 5b | Primary concerns and symptoms of the patient | Page 2 (Case Report, para 1) |
|  | 5c | Medical, family, and psycho-social history including relevant genetic information | Page 2 (Case Report, para 1) |
|  | 5d | Relevant past interventions with outcomes | Pages 2-3 (Case Report, para 2-3) |
| **Clinical Findings** | 6 | Describe significant physical examination (PE) and important clinical findings | Page 2 (Table 1) |
| **Timeline** | 7 | Historical and current information from this episode of care organized as a timeline | Figure 1 |
| **Diagnostic Assessment** | 8a | Diagnostic testing (such as PE, laboratory testing, imaging, surveys) | Pages 2-3, Tables 1-3 |
|  | 8b | Diagnostic challenges (such as access to testing, financial, or cultural) | N/A |
|  | 8c | Diagnosis (including other diagnoses considered) | Page 2 (Case Report, para 1) |
|  | 8d | Prognosis (such as staging in oncology) where applicable | Page 2 (Sokal score: low risk) |
| **Therapeutic Intervention** | 9a | Types of therapeutic intervention (such as pharmacologic, surgical, preventive, self-care) | Pages 2-3 (Case Report) |
|  | 9b | Administration of therapeutic intervention (such as dosage, strength, duration) | Pages 2-3 (imatinib 400mg, ponatinib 15-30mg, asciminib 40mg) |
|  | 9c | Changes in therapeutic intervention (with rationale) | Pages 2-3 (Case Report) |
| **Follow-up and Outcomes** | 10a | Clinician and patient-assessed outcomes (if available) | Page 3, Figure 1 |
|  | 10b | Important follow-up diagnostic and other test results | Page 3, Figure 1 (BCR::ABL1 monitoring) |
|  | 10c | Intervention adherence and tolerability (How was this assessed?) | Page 3 (no TKI withdrawal syndrome) |
|  | 10d | Adverse and unanticipated events | Page 2 (cerebral infarction with ponatinib) |
| **Discussion** | 11a | A scientific discussion of the strengths AND limitations associated with this case report | Pages 3-4 (Discussion, Limitations) |
|  | 11b | Discussion of the relevant medical literature with references | Pages 3-4 (Discussion) |
|  | 11c | The scientific rationale for any conclusions (including assessment of possible causes) | Pages 3-4 (Discussion) |
|  | 11d | The primary "take-away" lessons of this case report (without references) in a one paragraph conclusion | Page 4 (Conclusion) |
| **Patient Perspective** | 12 | The patient should share their perspective in one to two paragraphs on the treatment(s) they received | N/A |
| **Informed Consent** | 13 | Did the patient give informed consent? Please provide if requested | Page 4 (Declarations) - Yes |
